# Need for training or already OnTracc? Development and psychometric properties of an online transcultural competence questionnaire among psychotherapists

**DOI:** 10.3389/fpsyg.2022.1040626

**Published:** 2022-11-17

**Authors:** Marie-Christin Atzor, Katharina Piegenschke, Hanna Christiansen

**Affiliations:** ^1^Department of Psychology, Division of Clinical Psychology and Psychotherapy, Philipps-University of Marburg, Marburg, Germany; ^2^Department of Psychology, Division of Clinical Children and Adolescent Psychology, Philipps-University of Marburg, Marburg, Germany

**Keywords:** transcultural competence, online-questionnaire, psychotherapy, culturally diverse patients, OnTracc

## Abstract

The prevalence of mental disorders among people with migration experiences is high. Studies have shown that despite the increasing number of patients from different cultures requiring therapy, treating them is still a major challenge. Furthermore, professional training aimed at improving transcultural competence is rare, and psychometric instruments assessing transcultural competence require improvement. Accordingly, this study aimed to develop and evaluate an online transcultural competence questionnaire (OnTracc) to assess transcultural competence among psychotherapists. Based on extensive literature research and expert interviews, 38-self-report statements referring to aspects of transcultural therapy (e.g., skills, knowledge, and awareness) were compiled. In two studies, 306 psychotherapists completed the OnTracc questionnaire. The multicultural counseling inventory (MCI), personality factors (BFI-K), experience in transcultural therapy, and demographic data were assessed as control variables. The exploratory factor analysis (EFA) revealed a 3-factor structure. The reliability of the scale ranged from ω = 0.73 to 0.81. These scales demonstrated substantial convergent and discriminant validity with the BFI-K and MCI subscales. Factorial validity was confirmed through confirmatory factor analysis. This validated inventory is the first to assess transcultural competence in therapeutic settings in the German language. Further analysis of the factorial validity of the different samples is recommended. Given the increasing diversity in the therapeutic landscape, additional development could help close the gap between the evolving treatment demands of culturally diverse patients and the lack of consideration in mental health and education.

## Introduction

Cultural diversity has received increased attention in psychotherapy (Baruth, 2016; Von Lersner and Kizilhan, 2017) because of increasing immigration (International Organization for Migration, 2019; McAuliffe and Triandafyllidou, 2021). A special subgroup encountered by psychotherapists are refugees and asylum-seekers, as the number of refugees has considerably increased worldwide (UNHCR, the UN Refugee Agency, 2015; UNHCR, 2022), and these constitute a high-risk group for mental health problems (Heeren et al., 2014; Richter et al., 2015; Hajak et al., 2021).

Several studies have shown that refugee or asylum status influences health professionals’ treatment decisions (Drewniak et al., 2016; de Haan et al., 2018). Despite the increasing number of patients with migration experiences requiring therapy, treating culturally diverse patients remains a major challenge (Wohlfart et al., 2006; Mösko et al., 2013; Fauk et al., 2021). There is a reported significantly lower willingness of general practitioners to treat a person with refugee or asylum status in comparison to regular citizens or labor immigrants. Several challenges are known to play a key role in perceived barriers to client intake in diverse cultures (Drewniak et al., 2016; Fauk et al., 2021). To name only a few, most therapists reported a lack of knowledge, awareness, and sensitivity to cultural differences, fear of additional expenses (e.g., for interpreters), and additional work (Mösko et al., 2013) as substantial challenges in conducting psychotherapy.

Therefore, it seems reasonable to acquire and improve transcultural competence among psychotherapists and reduce fear and concerns about treating patients who have migrated through specific training programs. To develop training, it is necessary to define transcultural competence among psychotherapists and perform assessments using reliable and valid questionnaires. Feedback is valuable to researchers and educators for training evaluation. Therefore, a reliable and valid inventory is required. So far, there is no inventory assessing transcultural competence among psychotherapists and mental health workers developed in a German-speaking country. Developing such an inventory considering the German mental healthcare system is an important step in enabling self-assessment and reflection of transcultural competence among psychotherapists, deriving content-relevant training aspects, such as evaluating the effects of training, especially as Germany is an immigration site. Among countries, such as Turkey, Pakistan, Uganda, and Colombia, Germany is one of the main countries of arrival (UNHCR, 2022). In the year 2021, 27% of Germany’s population had experience with migration (Statistisches Bundesamt, 2022b). Between approximately 1 and 1.6 million people immigrate to Germany each year (Statistisches Bundesamt, 2022a,b). As we are currently facing the war in Ukraine, this number is expected to increase significantly in 2022, since more than 850.000 people fled from Ukraine to Germany between February and June 2022 (Statistisches Bundesamt, 2022a).

Thus, transcultural competence among psychotherapists is vital for the successful psychotherapeutic treatment of people with a migration background. This addresses the cultural components of psychotherapy and increases the effectiveness of therapy (Comas-Diaz, 2013; Sanchez et al., 2022). A meta-analysis (Tao et al., 2015) has shown that perceived multicultural competencies of therapists have an impact on therapy outcomes as well as on process measures such as a working alliance and client satisfaction. The main purpose of addressing transcultural competence among psychotherapists is to navigate culturally diverse values, beliefs, and behaviors to tailor psychotherapeutic care to meet patients’ unique social, cultural, and linguistic needs (DeAngelis, 2015; Raddawi, 2015). The American Psychiatric Association (American Psychological Association, 2003) stresses the need for cultural competency in their ethics codes and has adopted guidelines on multicultural training and research. Nevertheless, effective professional psychotherapeutic training aimed at improving transcultural competence is rare. Sue et al. (1982) developed the first well-established conceptualization of transcultural competence in psychology (Sue, 2001). Following this model, transcultural competence is based on three factors: (1) awareness of one’s personal beliefs, values, biases, and attitudes (awareness), (2) awareness and knowledge of the worldview of culturally diverse individuals and groups (knowledge), and (3) utilization of culturally appropriate intervention skills and strategies (skills; Sue, 2001). Awareness of attitudes/beliefs includes awareness of one’s cultural heritage and values, as well as biases that could impact the interaction with a culturally diverse patient. Knowledge refers to knowledge about a patient’s cultural background. Furthermore, it implies the knowledge of potential barriers within the institutional system one is working in and about aspects of migration processes. Contact-specific skills in the interaction with culturally diverse patients, for example, integrating the patients’ culture-specific explanatory model into the therapy. An all-encompassing conceptualization of transcultural competence is still being defined. Only within helping professions a recent review identified 35 definitions of cultural competence (Tehee et al., 2020). Furthermore, valid and reliable psychometric instruments assessing transcultural competence in psychotherapeutic care are lacking (Jager et al., 2021).

Some questionnaires have assessed cultural competence in culturally diverse environments (D’Andrea et al., 1991; Ponterotto et al., 1994; Kim et al., 2003). The Awareness, Skills, Knowledge: General (ASK-G; Domenech Rodríguez et al., 2022) is a newly developed self-report instrument based on the three factors of Sue (2001) to assess cultural competence within the general population, while the Multicultural Counseling Inventory (MCI; Sodowsky et al., 1994) are widely used self-report instruments to measure intercultural competence in counseling professions. The MCI was translated into German *via* the back-translation method (Brislin, 1970) and adapted linguistically from counseling to therapeutic settings. The MCI is based on the framework of Sue (2001) and captures four dimensions: multicultural counseling skills, multicultural awareness, multicultural counseling knowledge, and multicultural counseling relationships. These instruments show sufficient reliability, but their validity results are inconsistent (MCI; Ponterotto et al., 2002) and need further investigation (ASK-G; Domenech Rodríguez et al., 2022). Additionally, different subscales of existing questionnaires appear to have high interscale correlations. It is likely that the subscales are correlated in some way, however this high correlations between scales may indicate that there is inordinate overlap between the constructs that the subscales are attempting to measure (Martins, 2014). Furthermore, all available questionnaires have been developed in North America and thus refer to the local socio-cultural and political context (Dunn et al., 2006; Jager et al., 2021; Domenech Rodríguez et al., 2022). One available inventory in Germany is the cross-cultural competence instrument for the healthcare profession (CCCHP; Bernhard et al., 2015). This instrument specifically addresses general members of the healthcare system, rather than psychotherapists. It assesses transcultural competence in five dimensions: intercultural motivation, attitudes, skills, knowledge/awareness, and emotions/empathy. However, this has not yet been sufficiently evaluated. To our knowledge, there is no well-evaluated questionnaire that specifically assesses the transcultural competence of psychotherapists. The aim of the current study was to develop and evaluate a valid and reliable questionnaire assessing transcultural competence among psychotherapists working in mental healthcare in Germany. This tool should be applied as an evaluation tool for transcultural training, and to assess therapists’ needs and possible barriers in treating culturally diverse patients by supporting self-reflection. The theoretical framework of the questionnaire developed in this study is based on the three factors (awareness, knowledge, and skills) defined by Sue (2001) because of its well-established nature. First, an item pool was generated based on expert interviews. In the next step (Study I), the questionnaire was analyzed with an explorative approach to identify factors. The explanatory approach has been used to provide the factor-structure with the best fit since other measurements theoretically based on the three factors showed more than three factors in the scales (MCI, ASK-G). In the second study (Study II), the three-factor structure of the OnTracc questionnaire found in Study I was validated using a confirmatory approach.

## Study I methods

### Design and procedure

The current validation study addressed psychotherapists with all specializations and experience levels *via* an online survey. The survey was conducted on the UniPark platform (Questback (Unipark) GmbH, 2019). Data were collected from 18.05.2020 to 02.10.2020. Invitations were sent with detailed study information, as well as a link to the online survey. Licensed therapists and therapists in training were contacted *via* email lists of different German training institutions for psychotherapists and social media. Participants were allowed to start the survey after they provided informed consent. Besides the online transcultural competence questionnaire (OnTracc), participants were asked to fill out a socio-demographic questionnaire with 15 items in total and tools for validation, which are described in detail below. The expected time to complete the survey was 25 min. To increase the motivation to participate, free online training on transcultural competence was offered. Participation in the training was independent of the current study. The study was approved by the Ethical Committee of the Department of Psychology of Philipps University of Marburg (Reference: 2020-15 k).

### Development of the questionnaire OnTracc

The OnTracc was developed in several steps. First, an interview guide for exploratory expert interviews was developed. The goal of the interviews was to create an item pool based on the experience of therapists in transcultural psychotherapy. It included questions based on three factors of transcultural competencies (awareness of attitudes/beliefs, knowledge, and skills; Sue et al., 1992) and psychotherapeutic work. The final semi-structured interview consisted of 13 questions. Semi-structured exploratory interviews were conducted by the first and second authors with eight experts in the field of psychology and psychotherapy in August 2019. The duration of the interviews ranged from 12: 09 to 27: 47 (*M* = 20: 22 min, *SD* = 5: 16 min). Before the start of the interview, participants were informed about the research purpose, interview process, audio recording, and rights concerning participation, and were asked to provide written informed consent. The interviews were conducted at the University of Marburg, Germany. The answers were analyzed qualitatively and clustered. In the second step, we searched for additional items in the inventories described above to assess transcultural competencies. In the third step, the preliminary items were presented to 16 psychotherapists with different experience levels to critically evaluate their comprehensibility, redundancy, and relevance. This resulted in minor changes, especially concerning the wording. The final version of OnTracc consisted of 38 items rated on a five-point Likert scale ranging from (1) (*totally disagree*) to (5) (*totally agree*).

### Measurements

In addition to the OnTracc, further questionnaires were administered. Sociodemographic information (i.e., age, sex, educational level, and migration background), as well as details about the participants’ profession (i.e., training/license, duration of professional activity, number of culturally diverse patients in treatment, and workshop experience) were assessed. To assess the discriminant validity of transcultural competence, the survey also included the short version of the Big Five Inventory (BFI-K; Rammstedt and John, 2005), which measures the Big Five personality dimensions using 21 items in total, measured with a Likert scale from (1) (*totally disagree*) to (5) (*totally agree*). Rammstedt and John (2005) reported satisfactory values for the test and retest quality criteria (ω in the current study sample (openness, 0.63; consciousness, 0.68; extraversion, 0.85; agreeableness, 0.21; and neuroticism, 0.75)).

In this study, the convergent validity of the OnTracc questionnaire was investigated using the Multicultural Counseling Inventory (MCI; Sodowsky et al., 1994). It consists of 40 items rated on a four-point rating scale ranging from (1) (*very inaccurate*) to (4) (*very accurate*). It shows sufficient evidence for the skills (ω = 0.79), awareness (ω = 0.77), and knowledge scales (ω = 0.75) in terms of reliability and validity in the study sample.

### Statistical analyses

All analyses were performed using the IBM SPSS (IBM Corp., 2015; ver. 23.0), and RStudio (RStudio Team, 2020). Data were checked for outliers and extreme values. No influential values were excluded based on Cook’s distance measure (COO: Cook’s distance, Cook, 1979). For the analysis of sociodemographic data, frequencies or means and standard deviations were calculated.

Exploratory factor analysis was conducted to analyze the underlying dimensions of OnTracc (maximum likelihood analysis). All items were analyzed descriptively to determine their suitability for test construction.

We used oblique rotations (Promax) as we expected significant factor correlations. The Kaiser-Meyer-Olkin measure of sampling adequacy (KMO; Kaiser, 1960) was used to measure sample adequacy as well as the adequacy of individual variables. Bartlett’s test of sphericity was conducted to check whether the factor analysis was statistically justified. To identify the number of factors represented in the data, parallel analysis and a MAP test were conducted.

The eigenvalues of these factors were also considered. Items were excluded if they had factor loadings below λ = 0.30, following the guidelines of Worthington and Whittaker (2006), or multi-factorial loadings with a greater difference than d < 0.20 (*n* = 7). Furthermore, items with a very low commonality (h^2^ ≤ 0.13; *n* = 3) were excluded, as only a small part of the variance could be explained by the factors (Bühner, 2011). The exclusion of an item was rejected if content-related considerations argued against it.

Omega was calculated to examine reliability. This was done in response to the increasing criticism of the interpretability of Cronbach’s alpha in the literature (Peters, 2014; Hayes and Coutts, 2020) to adapt the statistical evaluation method to the current state of research. Furthermore, for the construct validity of OnTracc, Pearson’s correlations with MCI were calculated to investigate discriminant validity. For this, Pearson product–moment correlations between the factor-analytically determined scales of OnTracc and the scales of the BFI-K were calculated. Following the guidelines of Cohen (1988), correlation coefficients between 0.10 and < 0.30 were rated as small, between.30 and < 0.50 as medium and ≥ 0.50 as large effects.

### Sample characteristics

A detailed description of the samples is presented in Table 1. In the first survey, 134 participants completed OnTracc. The average time for completion was *M* = 30 m 17.37 s. The age of participants ranged from 24 to 79 years with an average of *M* = 36.01 (*SD* = 10.96) years. Most participants were female (75.4%), with 20.9% in total having a migration background. More than half of the participants were psychotherapists in training (57.0%). Medical psychotherapists and child and adolescent psychotherapists were represented only to a small extent in the sample. In total, 32.1% of the participants were licensed psychotherapists. Regarding the type of therapy practiced, cognitive behavioral therapy was indicated by most of the participants (72.4%), while 21.6% practiced psychodynamic psychotherapy, and 10.4% psychoanalytic psychotherapy. The vast majority of participants (88.1%) reported previous experience working with culturally diverse patients.

**Table 1:** Characteristics of sample 1 and sample 2.

	Study 1	Study 2	Test-statistics
n	134	172	
Age in years (M, SD)	36.01 (11.0)	39.67(13.0)	t (304) = −3.66**
Sex (% female)	75.4	83.1	χ^2^ (1) = 2.33
Migration background (%)	20.9	23.3	χ^2^ (1) = 0.24
Licensure (%)	32.1	55.8	χ^2^ (1) = 17.1***
Medical or psychological psychotherapist (%)			
Child and adolescent psychotherapist	3.0	8.1	χ^2^ (1) = 3.61
Child and adolescent psychotherapist in training	6.0	2.3	χ^2^ (1) = 2.66
Medical psychotherapist	3.0	3.5	χ^2^ (1) = 0.06
Medical psychotherapist in training	1.5	2.9	χ^2^ (1) = 0.67
Psychological psychotherapist	27.6	43.0	χ^2^ (1) = 7.74**
Psychological psychotherapist in training	57.0	40.1	χ^2^ (1) = 10.70**
Field of expertise (%)			
Cognitive behavioral therapy (CBT)	72.4	60.8	χ^2^ (1) = 0.25
Psychoanalytic psychotherapy	10.4	5.2	χ^2^ (1) = 0.09
Current workspace (%)			
Day-care hospital	1.5	2.9	χ^2^ (1) = 0.67
Psychiatric hospital	17.9	10.5	χ^2^ (1) = 3.53
Psychosomatic hospital	15.7	9.3	χ^2^ (1) = 2.88
Psychotherapy practice	31.1	50.0	χ^2^ (1) = 9.91**
Training Institute	50.7	26.7	χ^2^ (1) = 18.56***
University & Research	9,7	9.3	χ^2^ (1) = 0.01
Currently not therapeutically active	1.5	8.1	χ^2^ (1) = 6.72*
Further education on culturally-sensitive psychotherapy (%)	35.8	35.5	χ^2^ (1) < 0.01
Experience with culturally diverse patients (%)	88.1	87.2	χ^2^ (1) = 0.05
Transcultural supervision or intervision (%)	10.4	8.1	χ^2^ (1) = 0.10
Culturally diverse patients in treatment (M, SD)	1.99 (3.81)	4.42 (7.22)	t (282) = −3.49***
Experience abroad (more than 6 months %)	50.7	52.3	χ^2^ (1) = 0.08
Volunteering with refugees (%)	28.4	27.3	χ^2^ (1) = 0.04

N = sample size; M = mean; SD = standard deviation; t = t value; χ^2^ = Pearson’s chi-square; *p < 0.05, ** *p* < 0.01, ***p < 0.001 (two-sided).

## Study I results

### Results of exploratory factor analysis

The findings of the first study were as follows. As sample size is an important issue for factor analysis different guidelines are giving various recommendations (Taherdoost et al., 2022). Several guiding rules of thumb stated that small sample sizes are sufficient (Sapnas and Zeller, 2002; Kyriazos, 2018). A sample size of 134 was considered sufficient for conducting factor analysis. Since Bartlett’s test for sphericity was significant [*χ^2^* (378) = 1058.90, *p* < 0.001], the variables were considered to be correlated. The KMO value was.80 and thus was also considered appropriate for conducting a factor analysis (Kaiser, 1960). The measure of sample adequacy (MSA) was derived to determine whether the items were highly correlated. The correlation values of the individual items, with the remaining items, ranged from.57 to.88, and thus, all were above the established cut-off value of MSA.50. The requirements for conducting the factor analysis were met.

First, exploratory factor analysis was conducted to arrive at a statistical evaluation of the 38 items. They were examined in terms of distribution, item difficulty, and discriminatory power. Additionally, items were excluded from the final factor solution due to low factor loadings (λ < 0.30), low communalities, and high cross-loadings (> 0.03). In total, 10 items were excluded because of classified problematic parameters in conjunction with content plausibility.

Exploratory factor analysis was used to determine the number of factors to be extracted. Regarding the results of the MAP test (Velicer, 1976), scree plot (Cattell, 1966), and theoretical considerations of the construct of transcultural competence (Sue et al., 1982, 1992), the number of factors to be extracted was set at three. With this restriction, maximum-likelihood factor analysis was conducted. In total, 28 items remained, suppressing factor loadings of <0.30. This revealed an almost simple structure in which each item could be assigned to one of the three factors (Table 2). The number of factors of OnTracc explained 37.4% of the total variance.

**Table 2:** Factor loadings and commonalities of the explorative administrations of the OnTracc.

	Study I (N = 134)			
Items	Engagement	Awareness	Challenges	h^2^
OT_5	0.29			0.30
OT_6	0.30			0.20
OT_11	0.44			0.41
OT_16	0.83			0.55
OT_17	0.41			0.39
OT_23	0.43			0.27
OT_28	0.57			0.35
OT_9		0.40		0.21
OT_18		0.45		0.33
OT_19		0.42		0.39
OT_20		0.36		0.21
OT_24		0.30		0.15
OT_25		0.66		0.41
OT_31		0.52		0.33
OT_35		0.65		0.37
OT_36		0.90		0.65
OT_2			0.45	0.25
OT_3			0.40	0.29
OT_7			0.50	0.22
OT_8			0.66	0.37
OT_10			0.43	0.33
OT_12			0.31	0.07
OT_14			0.45	0.30
OT_21			0.33	0.13
OT_26			0.32	0.18
OT_33			0.40	0.29
OT_37			0.41	0.32
McDonald’s Omega ω	0.81	0.73	0.75	–

h^2^ = commonality. An overview of the German items and English translations can be found in (Supplementary Table A).

The first factor consisted of seven items that measured engagement in psychotherapy with culturally diverse patients. This includes working with a translator or engaging in migration-specific issues, more often beyond therapy and unpaid. Therefore, the first factor was named “Engagement,” which resulted in satisfactory internal consistency (ω = 0.81).

The second factor with 10 items assesses the extent to which one is aware of their own and the patient’s cultural background, for example, being aware of and integrating the patients’ culture-specific explanation model in psychoeducation. Thus, the factor was named “Awareness” and demonstrated sufficient internal consistency (ω = 0.73).

The third factor with 11 items measures challenges a therapist may experience concerning therapeutic work with culturally diverse patients, for example, finding it harder to work with culture-specific disease concepts and emotional handling of reports on war and torture. The third factor was called “Challenges,” and it had satisfactory internal consistency (ω = 0.75).

### Discriminant validity

Discriminant validity was identified by calculating the Pearson product–moment correlations between the defined scales of the OnTracc and BFI-K. Overall, small positive correlations were found with the extraversion, openness, and agreeableness scales (*r* = 0.17–0.29). Engagement was significantly correlated with extraversion (*r* = 0.25) and openness (*r* = 0.29) and negatively correlated with neuroticism (*r* = −0.17), as expected (Van Keuk et al., 2011). Challenges had a positive correlation with agreeableness (*r* = 0.26). Awareness was positively correlated with extraversion (*r* = 0.22) and openness (*r* = 0.19). A medium negative correlation of the OnTracc scale was found for the neuroticism scale (*r* = 0.16–0.36). The Conscientiousness scale of the BFI-K showed no significant correlation with the scales (for a detailed overview, see Table 3).

**Table 3:** Analysis of validity (Bivariate correlations of the OnTracc with the MCI and the BFI-K of Study I and II).

Study I	MCI^a^				BFI-K^b^				
	F 1	F 2	F 3	F 4	F 1E	F 2A	F 3C	F 4N	F 5O
Engagement	0.19*	0.68**	0.38**	0.43**	0.25**	0.12	0.02	−0.17*	0.29**
Awareness	0.35**	0.53**	0.61**	0.56**	0.22*	0.14	0.07	−0.16	0.19*
Challenges	0.40**	0.49**	0.37**	0.30**	0.19*	0.26**	0.14	−0.36**	0.20*
Study II									
Engagement	–	–	–	–	0.12	0.16*	0.09	−0.23**	0.27***
Awareness	–	–	–	–	0.23**	0.11	0.22**	−0.23**	0.42***
Challenges	–	–	–	–	0.14	0.12	0.21**	−0.24**	0.30***

^a^MCI Factor 1 = Multicultural Relationship, Factor 2 = Multicultural Awareness, Factor 3 = Multicultural Knowledge, Factor 4 = Multicultural Skills, ^b^BFI-Factor 1 = Extraversion, Factor 2 = Agreeableness, Factor 3 = Conscientiousness, Factor 4 = Neuroticism, Factor 5 = Openness to Experience, E = Item of the OnTracc-Engagement subscale, A = Item of the OnTracc-Awareness subscale, C = Item of the OnTracc subscale, * *p* < 0.05, ** *p* < 0.01, ***p < 0.001 (two-sided).

### Convergent validity

Regarding the convergent validity of OnTracc, positive and significant Pearson’s correlations with the MCI scales were found throughout, although the level of correlations varied between the individual scales. As Table 3 shows, all bivariate correlations between the individual scales of the two questionnaires were in the low-to-high range. Owing to the content similarity, this result was as expected. Awareness of OnTracc correlated highly with awareness, skill (MCI), and knowledge MCI scales, and OnTracc engagement correlated highly with Awareness of MCI.

In summary, we identified three subscales of the OnTracc questionnaire. The scales were named in terms of their content. Overall, the scales showed acceptable to good internal consistency and expected correlations with the MCI subscales, indicating convergent validity.

## Study II methods

### Study design and procedure

The aim of study II was to validate the three-factor structure of the OnTracc questionnaire as found in study I. Therefore, a confirmatory approach was used. Participants were recruited through social media (Facebook, Twitter, and Instagram) and mailing lists of educational institutions nationwide. Furthermore, clinical psychologists in private practice within Hessia were invited to participate. The primary eligibility criterion was that participants were psychotherapists or psychiatrists (licensed or in postgraduate training) for adults or children and adolescents. Data were collected from psychotherapists with all specializations and experience levels who were rewarded with online training in transcultural psychotherapy.

### Measurements

The collected sociodemographic and profession-related information corresponded to those of study I. Before completing the OnTracc, the BFI-K (Rammstedt and John, 2005) was used to examine discriminant validity. The reliability of the BFI-K scales in this study ranged between α = 0.49–0.76.

### Statistical analyses

All analyses were performed using the coding programs R (RStudio Team, 2020) and Amos (Amos, Version 23.0). ince existing questionnaires in the cultural field show moderate effects, we based our power calculations on a medium effect (*f* = 0.25, α = 0.05, power 95%; Lin et al., 2017; Benuto et al., 2018). Using G*Power (Faul et al., 2007), the minimum total sample size should be *N* = 74. As in Study I, frequencies or means and standard deviations were calculated for the analysis of sociodemographic data. The samples from Study 1 and Study 2 were compared using chi-square tests for frequencies and t-tests. First, the three-factor structure identified in Study I was tested by conducting a confirmatory factor analysis. To examine the multivariate normal distribution, the Mardia-Test (Mardia and Marshall, 1984) was used. A critical ratio (C.R.) ≥ 3.165 indicates a violation of the assumption of a multivariate normal distribution (Bühner, 2011). To estimate the model fit, the overall χ2-test of the hypothesized model, comparative fit index (CFI), root mean square residual (SRMR), and root mean square error of approximation (RMSEA) were used as goodness-of-fit indices. RMSEA values less than 0.08 show a good fit (Bühner, 2011), SRMR values lower than 0.11 and CFI values above 0.95, indicating a close fit to the suggested model (Bühner, 2011).

### Sample characteristics

The OnTracc was completed by 174 participants. Two participants were excluded because of conspicuous response patterns (answering every single item with 1). Thus, data with *N* = 172 were included in the analyses.

Participants were between 21 and 76 years of age (*M* = 39.67, *SD* = 13.00), with 83.1% female. The migration rate was reported as 23.3%. For detailed information as well as a comparison with the study sample, see Table 1.

Compared to the sample in Study I, the sample in Study II was significantly older [*t* (304) = −23.66, *p* = 0.009], and had more professional experience with culturally diverse patients [*t* (282) = −3.49, *p* = 0.001], and a higher percentage of licensed psychotherapists [*χ*^2^ (1) = 17.10, *p* < 0.001]. Furthermore, participants in Study II were more likely to work in their private practice [*χ^2^*(1) = 9.91, *p* = 0.002]. The samples did not differ from any other sociodemographic variables.

## Study II results

### Confirmatory factor analysis

**Figure 1:**
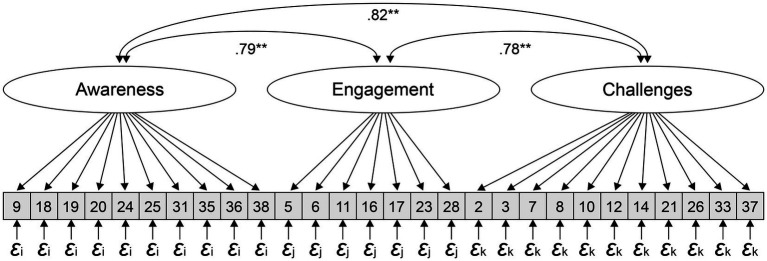
Three-factor model. 𝜺i = Error term of the 9 items of Awareness; 𝜺j = of the 7 items of Engagement 𝜺k = of the 11 items of Challenges. All parameter estimates were statistically significant (*p* < 0.001). Model Modifications: OT_35_t1_A ~ ~ OT_36_t1_A; OT_16_t1_E ~ ~ OT_28_t1_E; OT_19_t1_A ~ ~ OT_16_t1_E; OT_31_t1_A ~ ~ OT_37_t1_C; OT_23_t1_E ~ ~ OT_33_t1_C; OT_18_t1_A ~ ~ OT_17_t1_E; OT_38_t1_A ~ ~ OT_37_t1_C; OT_10_t1_C ~ ~ OT_26_t1_C; OT_31_t1_A ~ ~ OT_6_t1_E; OT_18_t1_A ~ ~ OT_19_t1_A; OT_31_t1_A ~ ~ OT_35_t1_A; OT_36_t1_A ~ ~ OT_2_t1_C; OT_18_t1_A ~ ~ OT_23_t1_E; OT_24_t1_A ~ ~ OT_28_t1_E; OT_25_t1_A ~ ~ OT_6_t1_E.

Initially, a three-factor solution, as suggested by the results of the EFA in Study I, was tested. Due to the relatively small sample of 172 participants, the parameters must be interpreted accordingly. In general, models with variables with high reliability may require smaller samples (Kyriazos, 2018). The parameter estimates of the final structural model are shown in Figure 1. Parameter constraints above a modification index of 7 were freely estimated so that the hypothesized CFA model included 15 additional path coefficients (Whittaker, 2012). This model fits the data reasonably well. Although *χ*^2^ was statistically significant (χ2 = 450.349, *p* < 0.001), other indices showed a good fit. RMSEA met the recommended criteria (RMSEA = 0.046), and CFI and TLI were above.90 (CFI = 0.914; TLI = 0.902). The SRMR was.065, well below the recommended.08 cutoff criteria, and the ratio of *χ*^2^ to its degrees of freedom was less than 2 (χ^2^/d. f. = 1.36). All parameter estimates were statistically significant (*p* < 0.001). Among other results, it is worth noting that demographic variables (e.g., age, sex, and job experience) did not improve the model fit.

Discriminant validity Pearson product–moment correlations between the OnTracc subscales and the BFI-K subscales were small to medium (−0.27 ≤ r ≤ 0.30, Table 3). As in Study I, OnTracc-Engagement correlated significantly with agreeableness, openness, and neuroticism (negative), but there was no correlation with extraversion in Study II. OnTracc-Awareness was significantly correlated with extraversion, conscientiousness, openness, and neuroticism (negative). OnTracc-Challenges correlated significantly with neuroticism (negative), conscientiousness, and openness.

### CFA model comparisons

To modify the model additional parameter constraints were set free between the manifest variables of the scales awareness, engagement, and challenges. In total 15 correlations of manifest variables were set free using the approach of modification indices. First, we tested whether a model with modifications had better fit than one without modifications and found that modifications showed significant improvement in model fit (*p* < 0.05). The model without modifications resulted in *χ^2^/df* = 0.92, RMSEA = 0.07, CFI = 0.78 and NFI = 0.63, and significantly higher values (AIC: 12593; BIC: 12779) than the model with modifications (AIC: 12428; BIC: 12665).

The model with modifications was also tested with and without covariances. Finally, the model with no added covariances, such as age, sex, and job experience, but with modifications revealed best fit-indices; *χ^2^/df* = 0.88, RMSEA = 0.05, CFI = 0.91 and NFI = 0.75. For an overview of the modified items see Figure 1.

## Discussion

The present study demonstrated the development and validation of the first inventory measuring transcultural competence in psychotherapists in Germany revealed solid psychometric properties. Hence, OnTracc is a promising tool to assess transcultural competence among psychotherapists and to evaluate the effectiveness of transcultural training in a first and to improve psychotherapy for culturally diverse patients in a second step.

The OnTracc showed good psychometric support for all three factors and 28 items. The findings demonstrated that the OnTracc measurement tool had good reliability in terms of internal consistency for all scales. These results were in line with the internal consistency of comparable tools, such as the MCI subscales, while some subscales of the CCCHP showed reliability coefficients even below acceptable levels (Bernhard et al., 2015).

To evaluate the validity of the OnTracc, we investigated the factorial, convergent and divergent validity. A three-factor structure was identified in the exploratory factor analysis. In accordance with similar inventories (CCCHP or MCI), the factor analysis of this study identified an “Awareness” factor, which was most strongly associated with the MCI’s knowledge scale. Since the knowledge scale has received most criticism because the knowledge of groups may mitigate the development of stereotypes (Beaulieu and Jimenez-Gomez, 2022), our items were not defined as knowledge about groups, but rather as a willingness to learn about cultures, so these items correlate with knowledge but are substantively matched with the “awareness of transcultural issues” scale. The other two factors identified were transcultural and therapy-related “challenges” and “engagement” with culturally diverse patients in therapy. The differences between the OnTracc factors (awareness, therapy-related challenges, and engagement), the tripartite model by Sue (2001; awareness, knowledge, and skills) and factors of others questionnaires such as the ASK-G (Domenech Rodríguez et al., 2022; awareness of self, awareness of others, proactive skills development and knowledge) might reflect the varying definition of cultural competence between and within disciplines (Tehee et al., 2020), the various facets of cultural competence and the resulting challenges in measuring cultural competences (Soto et al., 2018). The factor “therapy-related challenges” found in this study can be seen as a strength, since OnTracc claims to assess transcultural competence among psychotherapists, as conducting psychotherapy with culturally diverse patients requires partly different transcultural competence than transcultural interaction in other contexts.

Factorial validity was proved using confirmatory factor analysis. In a second step, statistical modifications were added to improve the model fit. Model-compliant paths with high correlations that made sense in terms of content were allowed. After modifying the items, the final model in Figure 1 adequately fitted the data, thus providing valuable information about the pattern of multivariate relationships among the items. Further, socio-demographic variables such as age, sex, educational level, and job experience did not improve the model fit.

Additional analysis of the model showed that “experience with culturally diverse patients” and “further education on the topic of transcultural psychology strengthens psychotherapists” engagement and awareness. As Barzykowski et al. (2019) suggested, mental health staff who interact with culturally diverse situations and who are open to learning more about transcultural issues might experience such encounters generally more positively, which is probably why they scored higher on the engagement and awareness scales in this study. These covariates have no impact on transcultural challenges, that might be perceived regardless of the experience level or continuing education. It might, therefore, be interesting to research the relationship between experience with diverse clients and transcultural competence. Thus, it is recommended that these covariates be considered when using OnTracc to assess transcultural competence.

Furthermore, results regarding convergent and discriminant validity could be obtained and interpreted to conceptualize the construct of transcultural competence more consistently and to distinguish it from other constructs in psychology. The convergent validity of OnTracc was supported by bivariate correlations between the individual scales of MCI and OnTracc. Regarding the BFI-K items, small to medium correlations with the OnTracc subscales supported the discriminant validity of the inventory. Since the CCCHP did not report convergent and discriminant validity, OnTracc was the only questionnaire developed in the German mental healthcare setting to assess these requirements.

Regarding personality (BFI-K), we found significant positive correlations between transcultural competence and personality traits, such as extraversion and openness. These results were consistent with the findings reported in previous studies (e.g., Barzykowski et al., 2019), and suggest that they may be related to transcultural competence. The findings of the present study support those of Rings and Allehyani (2020), who argue that openness may prevail in seeking experiences and interacting with patients from diverse cultures and may also increase their motivation to learn. This study shows that therapists with high extraversion and openness were more likely to voluntarily engage in transcultural encounters and may also be more willing to interact with culturally diverse patients when the opportunity arises. Such a constellation of personality traits (i.e., high extraversion and openness and low neuroticism) seems to be a predictor of high engagement and awareness in transcultural therapy settings. Both personality and seemingly general counseling competencies (Fuertes and Brobst, 2002) appear to have only small correlations between transcultural competencies, indicating that transcultural and general competencies overlap, but also have distinct effects (Sue and Zane, 1987). This again shows that the concept of transcultural competencies is interesting to grasp and provides valuable information. However, further investigations regarding convergent and discriminant validity are needed to advance research on the conceptualization of transcultural competence in psychotherapy, as there is an ongoing dispute about the meaning of the components (Kumaş-Tan et al., 2007; Tehee et al., 2020). Practitioners, educators, and researchers need to question and challenge these assumptions to understand, teach, practice, and evaluate transcultural competence.

### Limitations

The present study has some limitations. Its cross-sectional nature makes it extremely difficult to assess causality apart from theoretical considerations. Self-report bias (method variance), specifically transcultural competence, may occur (see also Benuto et al., 2019). With a multimodal approach, the overlap issue, as well as the influence of the social desirability of the self-report format, could be controlled (Dunn et al., 2006). In addition to OnTracc, a combination with other survey methods, such as external assessment tools, third-party rating procedures, or behavioral measures (Barzykowski et al., 2019), may further enhance validity.

Because we chose to limit the sample to psychotherapists, it is important to note that it is not yet known to what extent the results are generalizable to other professions in mental health care. Finally, the structural *a priori* model was not supported by the data and statistical modifications were required to achieve a reasonable model fit. Methodological deficiencies may also have played a role, as both study samples may not have been large enough and differ significantly regarding key sociodemographic characteristics, such as age and experience level, when treating culturally diverse patients. Therefore, more research is needed to cross-validate the model on another sample to avoid capitalization on chance (Döring and Bortz, 2016).

The questionnaire is the first attempt to highlight and psychometrically capture commonalities, in addition to differences between culturally diverse patients. Since the concept of transcultural psychotherapy with its numerous conceptualizations has yet to be debunked (Kocarek et al., 2001; Tehee et al., 2020), on the one hand, the questionnaire offers scope to further clarify and define the concept, and on the other hand, it is still a severe challenge of the entire field to narrow down the concept and make it measurable (Steinhäuser et al., 2014; Soto et al., 2018). Moreover, questionnaire items must be constantly updated in terms of political context and linguistic correctness.

### Advances and implications

Despite these limitations, the OnTracc can provide valuable insights for research purposes in evaluating transcultural training to increase transcultural competence in psychotherapy in the European context. To date, most models and tools have been developed in the United States, and there is still a lack of standardized, valid, and reliable instruments for psychotherapists (Barzykowski et al., 2019). So far, the evidence of improving transcultural competence through training is still at early stages, even if some promising results show the effectiveness of transcultural training for specific health workers, such as nursing students (Majda et al., 2021; Fadaeinia et al., 2022). As one reason is the difficulty of measuring cultural competence (Soto et al., 2018), the development of OnTracc was an important step to have a reliable and valid tool to measure the effectiveness of transcultural training among psychotherapists and to improve psychotherapy for culturally diverse patients in the future. OnTracc should be useful for researchers and educators to investigate the outcome of transcultural training for both short workshops and longer courses such as university classes. Further, OnTracc as a self-report tool can be used by psychotherapists to reflect on one’s own attitude and to become aware of one’s own cultural imprinting, and to assess intraindividual changes. The data of our two studies provided important information and empirical insights based on a transparent and systematic study design. To ensure the content validity of the OnTracc, a questionnaire was constructed based on the current literature (Asfaw et al., 2020) and theoretical models of transcultural competence (Sue et al., 1992). A qualitative study design preceded the study by conducting semi-structured in-depth expert interviews with psychotherapists working with culturally diverse patients in Germany. Thus, the items of the questionnaire present the main challenges in working with culturally diverse patients whereas high transcultural competence represents strategies they have found useful in dealing with these challenges.

Methodological strengths include the fact that our findings successfully confirmed OnTracc’s internal consistency consistently across the two studies. Furthermore, it should be emphasized that as a supplement to the calculation of Cronbach’s alpha, McDonald’s omega was determined as a measure of the internal consistency of the test scales to follow the current state of research, while previous studies used Cronbach’s alpha exclusively.

Within the framework of the investigations carried out, there is a need for further psychometric testing of the OnTracc, e.g., in terms of convergent and predictive validity With ASK-G (Domenech Rodríguez et al., 2022), a new tool to assess cultural competence with emphasis on race and ethnicity among the general population was published recently. As the authors are mentioning that ASK-G can be used by psychologists to evaluate transcultural competence in training and research, a future study should compare ASK-G and OnTracc in terms of validity such as variance explanation. The initial indications regarding the test-theoretical quality of OnTracc were determined.

## Conclusion

In conclusion, OnTracc is the first economical self-evaluation tool to assess transcultural competence in German-speaking psychotherapists. Despite these preliminary results in a still expanding field (Beaulieu and Jimenez-Gomez, 2022), the inventory demonstrated satisfactory psychometric properties in both studies. The associated scales of challenges to reduce fears and insecurities and to enhance engagement and awareness signified increased transcultural competence, which could be considered when starting to comfort practitioners to meet the increasing treatment needs of culturally diverse patients.

For practitioners, the OnTracc demonstrates high clinical relevance. It allows them to reflect on their own transcultural competence using the variety of items as a self-assessment. In training or supervision, the questionnaire can help to get a first impression of the extent to which transcultural competence can be promoted. Furthermore, it is possible to use the questionnaire as an external assessment tool for patients, who can evaluate their therapist on the basis of the items on transcultural competence. In the future, the questionnaire could be adapted for other healthcare domains (e.g., physicians, social workers). The number of culturally diverse patients continues to increase and validated psychometric instruments are lacking (Benuto et al., 2019). Therefore, culture sensitive mental healthcare in secondary education has received growing interest. Combined with further outcomes, the OnTracc questionnaire could contribute to the scientific evaluation of transcultural training.

## Data availability statement

The original contributions presented in the study are included in the article/Supplementary material; further inquiries can be directed to the corresponding author.

## Ethics statement

The studies involving human participants were reviewed and approved by the Ethical Committee of the Department of Psychology of Philipps University of Marburg (Reference: 2020-15 k). The patients/participants provided their written informed consent to participate.

## Author contributions

KP and M-CA contributed equally to the final version of the manuscript. HC helped to supervise and resource the project and revised it critically for important intellectual content and proofread several versions of the manuscript. All authors contributed to the article and approved the submitted version.

## Funding

Open Access funding provided by the Open Acess Publication Fund of Philipps-Universität Marburg with support of the Deutsche Forschungsgemeinschaft (DFG, German Research Foundation).

## Conflict of interest

The authors declare that the research was conducted in the absence of any commercial or financial relationships that could be construed as a potential conflict of interest.

## Publisher’s note

All claims expressed in this article are solely those of the authors and do not necessarily represent those of their affiliated organizations, or those of the publisher, the editors and the reviewers. Any product that may be evaluated in this article, or claim that may be made by its manufacturer, is not guaranteed or endorsed by the publisher.
