# Supplementary material for: Need for training or already OnTracc? Development and psychometric properties of an online transcultural competence questionnaire among psychotherapists
Source: Front Psychol. 2022 Nov 17;13:1040626. doi: 10.3389/fpsyg.2022.1040626 (PMC9712191; doi:10.3389/fpsyg.2022.1040626)
Supplement: Supplementary file 1 [file Table_1.DOCX]

Supplementary Material

**Supplementary Table A** Items of the OnTracc and their English translations.

| Item Number | German Items | English Translation |
| --- | --- | --- |
| E_1 | Ich habe genügend Kenntnisse über die migrationsspezifischen rechtlichen Rahmenbedingungen (z.B. Aufenthaltsstatus, Asylrecht). | I have enough knowledge about migration-specific legal frameworks (e.g. resident status, right of asylum) |
| E_2 | Ich engagiere mich aktiv für den Abbau von Barrieren für Menschen mit Flucht- und Migrationshintergrund in der Psychotherapie (z.B. gegen Mangel an Sprachmittlern ...). | I am actively involved in the process of reducing barriers for refugees and people with a migration background in accessing psychotherapy (e.g. against the shortage of translators). |
| E_3 | Falls notwendig, bin ich bereit (auch unbezahlt) einen Mehraufwand für Patient*innen mit Flucht- und Migrationshintergrund zu betreiben. | I am ready for additional expenses (even without extra payment) to support my patients who had to flee and have a migration background. |
| E_4 | Wenn ich im Ausland bin, versuche ich Zeit mit Einheimischen zu verbringen | If I am in a foreign country, I try to spend time with the locals. |
| E_5 | Ich traue mir zu mit einem*r Dolmetscher*in zu arbeiten (bzw. habe dies bereits). | I am confident in working with translators (or have had experiences already). |
| E_6 | Wenn ich das Gefühl habe, dass der Dolmetscher*in nicht korrekt übersetzt (z.B. weniger/ etwas Anderes/mehr sagt), traue ich mir zu, die Therapie zu unterbrechen und den Dolmetscher*in an die vereinbarten Instruktionen zu erinnern. | If I think the translator is not translating correctly, I have the confidence to interrupt the therapeutic session and remind the translator of the agreed guidelines. |
| E_7 | Ich erfrage die sozio-politischen Hintergründe meiner Patient*innen | I ask my patients about their socio-political background. |
| A_1 | Ich integriere das kulturell geprägte Erklärungsmodell meiner Patient*innen in die Therapie. | I integrate my patients’ culturally shaped explanation model into therapy. |
| A_2 | Bei Bedarf beziehe ich alternative Erklärungsmodelle (z.B. Glaube an ein vorbestimmtes Schicksal, Magie etc.) aktiv in die Therapie ein. | I actively include alternative explanation models (e.g. belief in destiny, faith in magic) into therapy if required. |
| A_3 | Ich traue mich nachzufragen, wenn ich im transkulturellen Setting etwas nicht verstehe. | I am confident to ask if I fail to understand something within the transcultural setting. |
| A_4 | Ich berücksichtige die kulturell bedingten emotionalen und somatischen Unterschiede in der Symptombeschreibung. | I take culturally-related differences in symptom description into account. |
| A_5 | Ich informiere mich über kulturelle Gegebenheiten: (religiöse) Feste und Feiertage, die die Therapie beeinflussen könnten. | I learn about cultural aspects that may influence the therapeutic process (religious and public holidays). |
| A_6 | Ich bin sensibilisiert für kulturelle Barrieren im Hinblick auf eine Psychotherapie. | I am sensitized to cultural barriers referring to psychotherapy. |
| A_7 | Wenn ich unabsichtlich eine kulturelle Regel verletze spreche ich dies an. | If I break a cultural rule unintentionally, I will address it. |
| A_8 | Ich bin mir über meine eigene kulturelle Prägung bewusst (Werte, Normen, Tabus, etc.). | I am aware of my cultural background (values, norms, taboos, etc.). |
| A_9 | Ich bin sensibilisiert für die Verwendung und Interpretation von nonverbalen Äußerungen. | I am sensitized to the use and interpretation of nonverbal communication. |
| C_1 | Es kommt vor, dass Menschen mit Fluchthintergrund während der Therapie abgeschoben werden - Trotz des Wissens über eine mögliche Abschiebung kann ich weiterhin gut mit der*m Patient*in arbeiten. | Refugees may be deported to their countries of origin during the therapeutic process. Despite knowing about this possibility, I can still work well with the patients. |
| C_2 | Berichte von Krieg und Folter belasten mich persönlich nicht mehr oder weniger als andere traumatische Erfahrungen. | Reports on war or torture impact me in the same way as reports on other traumatic events. |
| C_3 | Wenn mein*e Patient*in eine andere Hautfarbe hat als ich, dann beeinflusst mich das nicht. | If my patients have a different skin color than me, it will not influence me. |
| C_4 | Wenn ich kulturelle Vorurteile bei mir bemerke, gelingt es mir gut, sie in der Therapie auszublenden. | If I notice cultural biases within myself, I manage to block them out easily during therapy sessions. |
| C_5 | Es kommt vor, dass in der Therapie noch weitere Anforderungen an mich gestellt werden (z.B. Familienmitglieder, Freunde wollen auch Hilfe). Mir gelingt es gut, mich davon zu distanzieren | If patients have additional requests during the therapeutic process (e.g. family members or friends also ask for help), I can distance myself from it. |
| C_6 | Es fällt mir leicht, mich in die Krankheitsmodelle von Patient*innen aus anderen Kulturen reinzudenken. | For me, it is easy to deal with the disease model of patients with different cultural backgrounds. |
| C_7* | Ich befürchte, dass ich mit geflüchteten Patient*innen nicht gut arbeiten zu können, da sie ein anderes Verständnis von Krankheit und Psyche haben. | I worry about my skills to work with refugees because they have a different understanding of diseases and mental health. |
| C_8 | In der Arbeit mit Menschen mit Flucht- und Migrationshintergrund nehme ich neben Unterschieden auch Gemeinsamkeiten zwischen uns wahr. | Besides differences, I also perceive similarities between my patients and me, when I work with refugees or people with a migration background. |
| C_9 | Mir gelingt es, auch über kulturelle Unterschiede hinweg eine gute therapeutische Beziehung zu meinen Patient*innen aufzubauen. | I succeed in building good therapeutic relationships with my patients despite cultural differences. |
| C_10 | Ich bin bereit meine Therapiemethoden an die Gegebenheiten der transkulturellen Psychotherapie anzupassen. | I am willing to adapt my therapeutic methods to the circumstances of transcultural psychotherapy. |
| C_11* | Ich vermeide transkulturelle Therapie, weil ich dabei auf den Kosten sitzen bleiben könnte. | I am avoiding transcultural therapy because I may bear all the costs myself. |

Notes. E = Item of the subscale OnTracc-Engagement, A = Item of the subscale OnTracc-Awareness, C = Item of the subscale OnTracc- Challenges,* Reversed item.
